# Comparing zirconia to anterior strip crowns in primary anterior teeth in children: a randomized clinical trial

**DOI:** 10.1186/s12903-020-01305-1

**Published:** 2020-11-10

**Authors:** Sumer M. Alaki, Bashaer S. Abdulhadi, Medhat A. AbdElBaki, Najlaa M. Alamoudi

**Affiliations:** Faculty of Dentistry, King Abdulaziz University, P. O. Box 1970, Jeddah, 21949 Kingdom of Saudi Arabia

**Keywords:** Zirconia crowns, Primary anterior teeth, Strip crowns, Multi-surface dental caries, Full coverage, Anterior restorations

## Abstract

**Background:**

Providing restorations to anterior teeth in children is a challenging task due to the need for high esthetics, strength, and durability. This study was done to compare prefabricated primary zirconia with resin composite strip crowns on primary maxillary central and lateral incisors with regards to gingival health, plaque accumulation, recurrent caries, restoration failure, and opposing teeth wear over a period of 3, 6 and 12 months.

**Methods:**

Children attending the King Abdulaziz University, Faculty of Dentistry (KAUFD) clinics who needed restorations were screened for inclusion criteria. A total of 120 teeth were treated; 60 with zirconia and 60 with strip crowns. Randomization was done by simple random allocation using SPSS software version 20.0 (Armonk, NY; IBM Corp.). A simple descriptive statistic was used for analysis by Wilcoxon Signed-Rank test and Chi-square test. Level of significance was set at (α = 0.05) and level of confidence at (95%). The presented research was registered retrospectively at ClinicalTrials.gov in 6th of August 2017, under registration number NCT03184012.

**Results:**

Zirconia crowns showed significantly less gingival bleeding at the 3- and 6-months follow up periods (*p* < 0.006, *p* < 0.001; respectively), less plaque accumulation at all follow up visits (*p* < 0.001), no restoration failure (*p* < 0.001), but more wear to opposing teeth (*p* < 0.02). No significant difference was found between the two crowns with regards to recurrent caries (*p* < 0.135).

**Conclusion:**

Based on our data we conclude that overtime teeth covered with zirconia crowns show better gingival health and less bleeding, plaque accumulation as well as less loss of material. On the other hand, zirconia can cause more loss of opposing tooth structure.

## Background

Early childhood caries (ECC) is a chronic multifactorial disorder which continues to be dominant in children, especially in families of low socioeconomic status [1–7]. Early childhood caries is defined as “the existence of one or more tooth decays (non-cavitated or cavitated lesions), missing (due to caries), or filled tooth surfaces in any primary dentition of children under the age of six years” [1]. Severe early childhood caries (S-ECC) is a progressive carious form in children, categorized in accordance to the number of affected teeth and the age of patient. The presence of smooth surface caries is considered to be an indication of S-ECC in patients below three years of age [8]. In children between three to five, S-ECC is defined as “one or more cavitated, missing (due to caries), or filled smooth surfaces in primary maxillary anterior teeth or a decayed, missing, or filled score of greater than or equal to four (age three), greater than or equal to five (age four), or greater than or equal to six (age five) surfaces” [1].

Providing care to children who are considered to be at risk from ECC, can be achieved by a specialist who received adequate training and has experience in treating children as well as disease process [1]. To perform treatment safely, effectively, and efficiently, the pediatric dentist can use behavior guidance techniques, protective stabilization and/or sedation or treatment under general anesthesia [1].

Despite their poor esthetics stainless steel crowns (SSC) are often the treatment of choice for multi-surface carious teeth and lesions with widespread white spots. Primary incisors with large or multi-surface caries can be restored with resin composite strip crowns if there is sufficient tooth structure after removing all carious tissues [9]. When remaining tooth structure is minimal and not enough for bonding, pre-veneered aesthetic crowns is a favorable solution. Stainless steel crowns with cosmetic facing have good aesthetics and good retention even with minimal remaining tooth structure. Moisture and hemorrhage control are not critical with these restorations which need minimal chair time and offer full coverage and protection.

More recently, zirconia aesthetic crowns for pediatric patients appeared in the market. Zirconia is a crystal-like dioxide of zirconium that possess a metal like mechanical properties and a tooth like color, and the ready to use zirconia crowns are available for primary teeth. Although there is high acceptance of zirconia crowns, the literature lacks solid proof for their pediatric clinical performance [32]. There are limited clinical studies that are currently ongoing, however until the outcomes of adequate number of prospective clinical trials with enough long-term follow-up periods is available evidence to ensure clinical success and durability of these crowns are leftover uncertain [33].

This study was aimed at comparing prefabricated primary zirconia with resin composite strip crowns on primary maxillary central and lateral incisors with regards to gingival health, plaque accumulation, recurrent caries, restoration failure, and opposing teeth wear over a period of 3, 6 and 12 months.

## Methods

### Sample size calculation

G*Power 3.1.9.2 software (Franz Faul, Universität Kiel, Germany, 2014) performed for power analysis, indicated that we needed a total 120 teeth (corresponding to around 30 children to achieve 80% power with 95% confidence assuming medium effect size in the mean change in gingival health 6 months after crown application in the zirconia and composite groups with the assumption of non-normal distribution. The number of crowns in each arm will be 60.

### Sample selection

A sample of 120 primary upper anterior incisors was treated in the Pediatric Dental Clinics, King Abdulaziz University, Faculty of Dentistry (KAUFD), at Jeddah, Saudi Arabia. (32 patients, 28 of them restorations were obtained to upper 4 anterior teeth and 4 of them restorations were done to upper tow central anterior teeth). All children visiting the clinics between November 1, 2015 to January 31, 2016 with the following criteria were included in the study:

1. Healthy four to six years old children.
2. Those having opposed anterior teeth.
3. No history of systemic illness or dental developmental anomalies which can affect dietary patterns, caries susceptibility or the selection of restorative materials to the best of current knowledge.
4. Minimal of two surfaces of caries in the targeted teeth.
5. Patient with Early Childhood Caries as defined by AAPD, 2016.
6. Cooperative patients who had behavioral rating “positive” or “definitely positive” followed the Frankl behavior classification scale [10].

Written consents were obtained from the parents/guardians after explaining the full details of the treatment procedure and its possible outcomes, discomfort, risks, and benefits.

No patient was excluded based on gender, race, social or economic background. Oral hygiene instructions were given and reinforced in each follow up visit.

### Exclusion criteria

Children having the following criteria were excluded from this research:

1. Teeth with proximity to exfoliation and resorption of the root passed its half.
2. Presence of single surface caries not involving the proximal surfaces.
3. Teeth that have been subjected to trauma.
4. Anxiety and lack of cooperation which required treatment under general anesthesia.
5. Bruxism.
6. Special health needs.
7. Presences of teeth wear on the opposing teeth, or absence of opposing. (See Flow Chart)

### Study design

The present study was a randomized controlled clinical trial that followed the guidelines published by Consolidated Standards of Reporting Trials (CONSORT) [11]. This research has been ethically accepted by the Committee of Research Scientific Unit at King Abdulaziz University with reference no. 076-16. Before enrolment, every patient’s parent/ guardian signed an informed consent sheet. The parameters that were evaluated in this study are listed in Table 1. Also, the presented research was registered at ClinicalTrials.gov under registration number NCT03184012.

**Table 1:** The description of the criteria used to record the clinical parameters

Criteria	Score	Description
Gingival Health	Alpha	No gingival bleeding
	Bravo	Bleeding with probe
	Charlie	Spontaneous bleeding
Plaque index*	0	No plaque
	1	A film of plaque adhering to the free gingival margin cannot be seen with the naked eye. But only by using disclosing solution or by using probe
	2	Moderate accumulation of deposits within the gingival pocket, on the gingival margin and/ or adjacent tooth surface, seen by naked eye
	3	Abundance of soft matter within the gingival pocket and/or on the tooth and gingival margin
Secondary caries**	Alpha	No caries present
	Charlie	Caries present
Restoration failure**	Alpha	Crown appears normal, no cracks, chips, or fracture
	Bravo	Small but noticeable area of loss of material
	Charlie	Large loss of crown material
	Delta	Complete loss of crown
Proximal contact**	Alpha	Resistance met when passing floss
	Bravo	Floss passed without resistance but contact present
	Charlie	No contact with adjacent tooth
Marginal integrity**	Alpha	Close marginal adaptation
	Bravo	No detectable margin
	Charlie	Detectable margin
Occlusion**	Alpha	Normal occlusion
	Charlie	Faulty occlusion
	Alpha	Normal occlusion
*Tooth wear of opposing*		
Teeth***	0	No loss of enamel surface characteristics, no loss of contour
	1	Loss of enamel surface characteristics, minimal loss of contour
	2	Loss of enamel exposing dentine for less than one third of surface, loss of enamel just exposing dentin, defect less than 1 mm deep
	3	Loss of enamel exposing dentin for more than one third of surface, loss of enamel and substantial loss of dentin, defect less than 1–2 mm deep
	4	Complete enamel loss, pulp exposure, secondary dentin exposure, pulp exposure or exposure of secondary dentin, defect more than 2 mm deep, pulp exposure, secondary dentin exposure

^*^Silness and Löe criteria (Loe, H., 1967)

^**^US Public Health Service “USPHS”, Alpha criteria rating system (Ryge, 1980)

^***^Smith and Knight Tooth Wear Index (Bardsley, 2008; Smith and Knight, 1984)

### Randomization

Concealment was applied for randomization of children in each group to either resin composite strip crown or zirconia crowns. Randomization was done using SPSS software version 20.0 (IBM Corp., Armonk, NY) Allocation concealment was assured by handling sequentially numbered opaque sealed envelopes to the attending candidate. At the time of crown application, an envelope was opened and allocate the child to the written restoration material inside that envelop.

### Examiner calibration

The same examiner was responsible of preparation and evaluation. To test the reliability of the examiner and evaluate the restoration performance, sixteen teeth were treated by full coronal restorations then examined. The examiner evaluated those teeth based on the standard evaluation criteria of the study. The results produced by the examiner were analyzed using Cohen’s kappa to test intra-examiner reliability.

### Procedure

**Fig. 1:**
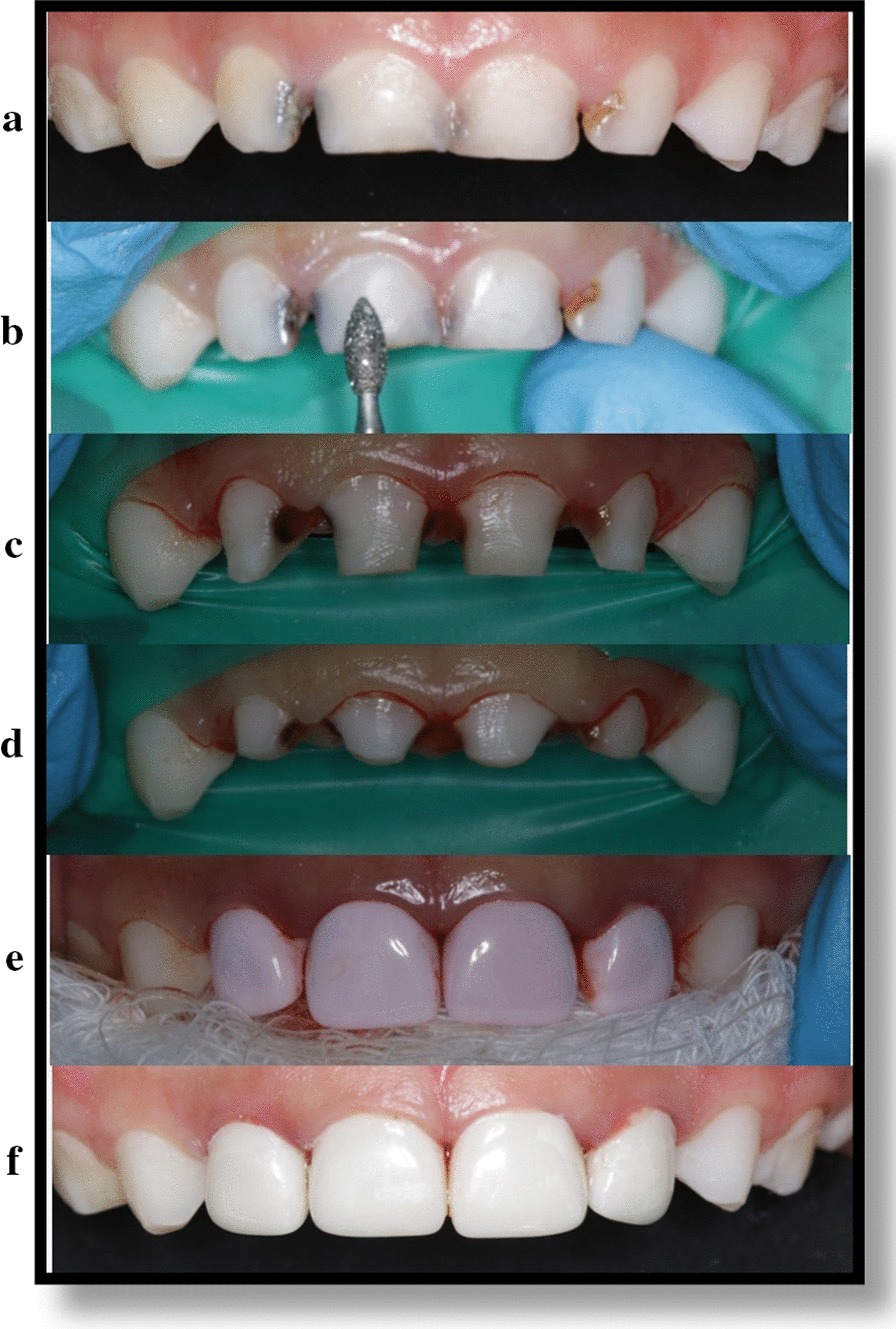
Anterior zirconia crown’s application technique. **a** Pre-operative teeth’s picture, **b** Facial reduction, **c** Interproximal reduction, **d** Incisal reduction and completion of caries removal, **e** Pink-crown try in, **f** After Cementation
(B) *Strip crowns* Prior to treatment appointments, strip crowns were prepared and adjusted. Escape venting holes were prepared by piercing the mesial or distal incisal angles of the crowns, this was achieved using sharp explorers (this hole formed a core vent to allow extra air bubbles trapped inside the crown to go out easily). Strip crowns were seated and fitted after applying a coating of resin-modified glass ionomer base to protect dentinal tissues. Each crown was cured individually after filling with composite resin as the adjacent strip crowns placed (unfilled) on their respective teeth to make sure that appropriate spacing between crowns preserved. A cleoid/discoid carver or scalpel was used to peel off the strip crown shell from the lingual side then ooclusion is checked and adjusted if needed (Fig. 2).

All treatments were done under nitrous oxide sedation and physical restrains to manage the children behavior, topical anesthesia as well as local infiltration was administered. The following clinical procedures were done in each group:

(A) *Zirconia crowns* The crowns were selected previously based on the mesio-distal measurement of the tooth. Each tooth was prepared for “passive fit” as the zirconia crowns are not flexible. According to the manufacturer instructions, incisal edge was reduced to obtain a 2 mm clearance. For the labial surface: the 2-plane reduction was made close to natural tooth and for proximal surface the distance to the adjacent teeth was considered and parallel mesial and distal walls were created extending 1–2 mm subgingivally. Enough reduction of cingulum was done on the palatal surface. Feather-edge margins were provided about 1–2 mm subgingivally. After evaluation made for marginal fit, the zirconia crowns were cemented with light cure resin cement (NuSmile BioCem®) and firm consistent pressure at proper position was applied on the tooth till the initial set (Fig. 1).

**Fig. 2:**
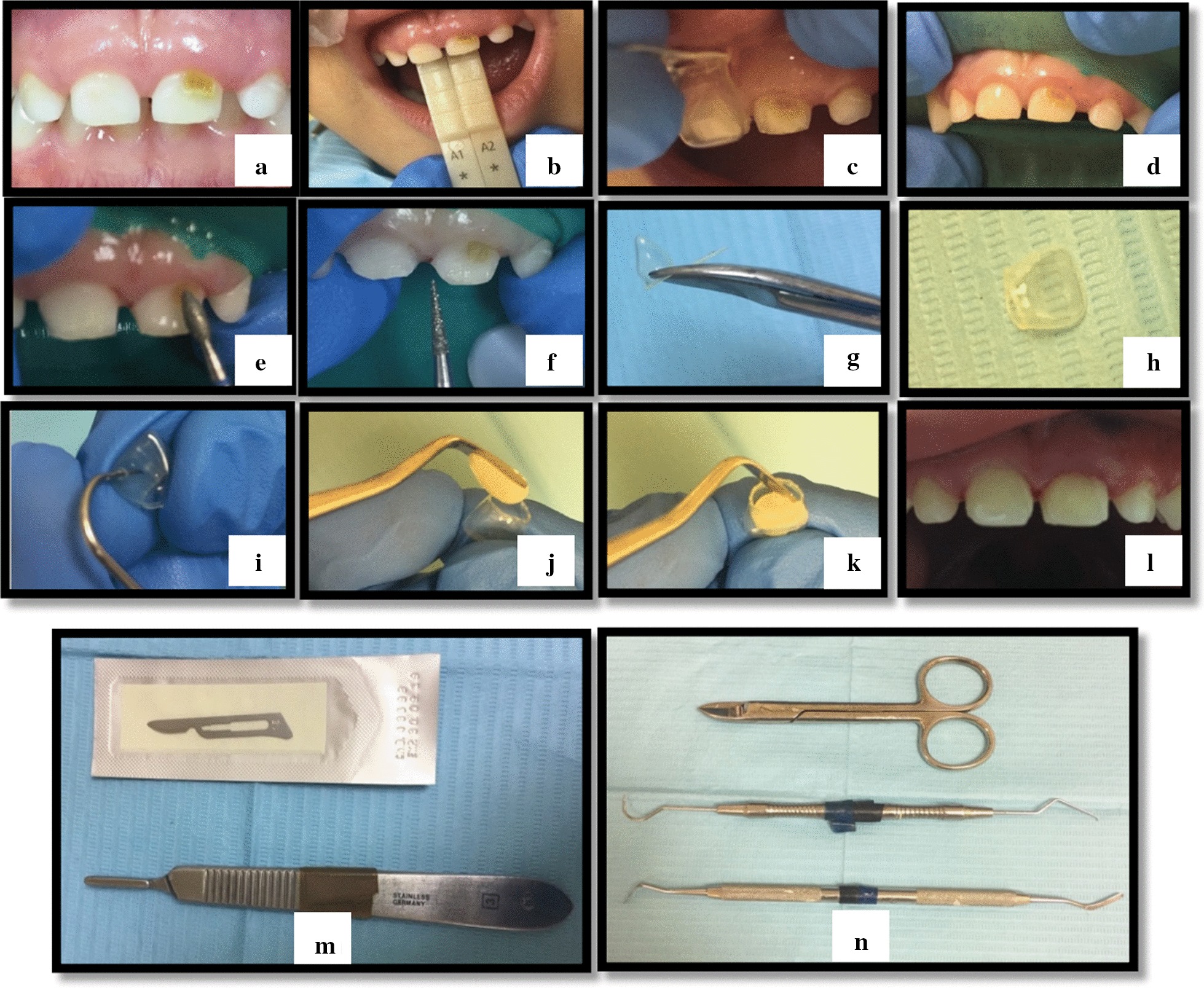
Anterior strip crown’s application technique. **a** Pre-operative teeth’s picture, **b** Shade selection, **c** Crown selection prior to tooth preparation, **d** rubber dam application, **e** Incisal, facial and lingual tooth reduction using football diamond bur, **f** Proximal tooth reduction using fine tapered diamond bur. **g** Cutting the strip crown using Curved Crown Scissor. **h** Strip crown after cutting. **i** Hole piercing in the lingual side of the crown using dental probe. **j** Applying resin composite material (Z100) into the strip crown using composite spatula. **k** Adaptation of composite material to the sides of the strip crown. **l** Teeth after application of strip crowns. **m** Scalpel used to peel off the strip crowns from the teeth after light cure. **n** Instrument used in the procedure from top: Curved Crown Scissor, dental probe, composite spatula

### Statistical analysis

This study was analyzed using IBM SPSS version 23 (IBM Corp., Armonk, NY). Simple descriptive statistics were applied to characterize the variables of the study via tally and percentages for the definitive and nominal variables, whereas mean and standard deviations were used to represent the constant variable. In comparing the distributions of two variables, a *chi-square test* were used. These tests were assumed to be observing normal distribution. Friedman analysis was used to check the difference of Gingival Health, Plaque index, Recurrent caries, Restoration failure, Tooth wear of opposing relative to multiple time points and separated by crown types. These tests were done with the assumption of normal distribution.

## Results

The intra-rater agreement and correlation of the examiner was calculated using Cohen’s Kappa Co-efficient and the clinical calibration results produced by the examiner were found to be at an excellent agreement range (0.97).

1. *Proportion distribution of demographic data*
  Full coronal restorations were placed on 120 primary maxillary anterior teeth (64centralincisors,56lateralincisors) on 32 patients, (44malesand76female). The mean age of patients at the base line was 4.43 years, with no drop out until the 12 months follow up. (Table 2).
2. *Gingival health evaluation*
  Gingival health as measured by bleeding with probing is depicted in Table 3. It can be seen that at the 3-months follow-up significantly more teeth in the strip crown group were bleeding compared to the zirconia groups (*p* < 0.006). At the 6-months follow-up also more teeth in the strip crown group were bleeding (*p* < 0.001). However, at the last follow-up visit at 12 months both groups showed no bleeding.
3. *Plaque index evaluation*
  Both groups were examined for plaque indices and scored according to Silness and Löe criteria. at the beginning of the study dental prophylaxis was provided to all patients and oral hygiene instructions were given to patients and their parents as well. Table 4 shows plaque indices at the follow-up visits. During the 3-months follow up more zirconia covered teeth showed either no plaque, or less plaque accumulation compared to teeth covered with strip crowns (*p* < 0.001). At the 6-months follow-up also more zirconia covered teeth showed less plaque accumulation (*p* < 0.001). At the end of the study all teeth covered with zirconia were free of plaque while only 80% of those covered with strip crowns were free of plaque and 20% had a film of plaque at the gingival margin (*p* < 0.001).
4. *Secondary caries evaluation*
  Evaluation of recurrent caries was done by visual inspection according to modified United States Public Health Service (USPHS) criteria.[12] The results showed that none of the teeth covered with zirconia crowns developed caries during the entire period of follow up. On the other hand, teeth that received strip crowns restorations had no recurrent lesions in the 3- and 6-months follow up but when it reached the 12-months follow up, 6.7% presented with recurrent caries. This finding, however, was not significant by applying *Chi-Square Test (p* < 0.135*)* (Table 5).
5. *Restoration failure evaluation*

**Table 2:** Proportion distribution of demographic data

Demographics	N	Min	Max	Mean	SD
Age	32	3.0	5.5	4.43	0.7

		Count	%		
Total		32	100.0		
Nationality	Saudi	25	78.1		
	Non-Saudi	7	21.9		
Gender	Male	12	37.5		
	Female	20	62.5		
Restoration type	Zirconia crown	60	50.0		
	Strip crown	60	50.0

**Table 3:** Gingival health evaluation

Chi-square test	Zirconia crowns	Strip crowns	*p*-value
*Gingival Health (assessed as bleeding on probing)*			
At 3 months	24 (40.0%)	40 (66.7%)	0.006^a^
At 6 months	0 (100.0%)	28 (46.7%)	< 0.001^a^
At 12 moths	0 (100%)	0 (100%)	N/A^c^

Friedman test	Mean rank		
	Total cases	Zirconia crown	Strip crown
Baseline	3.61	3.80	3.43
At 3 months	2.68	2.59	2.77
At 6 months	2.09	1.80	2.37
At 12 moths	1.61	1.80	1.43
p-value	< 0.001^b^	< 0.001^b^	< 0.001^b^

^a^Significant using Chi-Square Test @ < 0.05 level

^b^Significant using Friedman Test @ < 0.05 level

^c^No statistics are computed because variable is a constant

**Table 4:** Plaque Index evaluation

	Chi-square test	Zirconia crowns	Strip crowns	*p*-value
*Plaque index*				
At 3 months	No plaque	20 (33.3%)	4 (6.7%)	< 0.001^a^
	A film of plaque adhering to the free gingival margin, cannot be seen with the naked eye	36 (60.0%)	44 (73.3%)	
	Moderate accumulation of deposits in the gingival pocket, on gingival margin and/or adjacent tooth surface, can be seen by the naked eye	4 (6.7%)	12 (20.0%)	
At 6 months	No plaque	47 (79.7%)	24 (40.0%)	< 0.001^a^
	A film of plaque adhering to free gingival margin, cannot be seen by naked eye	12 (20.3%)	36 (60.0%)	
At 12 months	No plaque	58 (100.0%)	48 (80.0%)	0.001^a^
	Film of plaque adhering to the free gingival margin, cannot be seen with naked eye	0 (0.0%)	12 (20.0%)	

Friedman test	Mean rank			
	Total cases	Zirconia crown	Strip crown	
Baseline	1.85	2.07	1.63	
At 3 months	3.56	3.45	3.67	
At 6 months	2.58	2.41	2.73	
At 12 moths	2.02	2.07	1.97	
p-value	< 0.001^b^	< 0.001^b^	< 0.001^b^	

^a^Significant using Chi-Square Test @ < 0.05 level

^b^Significant using Friedman Test @ < 0.05 level

**Table 5:** Secondary caries evaluation

Chi-square test		Zirconia crowns	Strip crowns	*p*-value
*Recurrent caries*				
At 3 months	No caries present	60 (100.0%)	60 (100.0%)	N/A^b^
At 6 months	No caries present	59 (100.0%)	60 (100.0%)	N/A^b^
At 12 months	No caries present	58 (100.0%)	56 (93.3%)	0.135
	Caries present	0 (0.0%)	4 (6.7%)	

Friedman test	Mean rank			
	Total cases	Zirconia crown	Strip crown	
Baseline	2.48	2.50	2.47	
At 3 months	2.48	2.50	2.47	
At 6 months	2.48	2.50	2.47	
At 12 moths	2.55	2.50	2.60	
p-value	0.007^a^	N/A^b^	0.007^a^	

^a^Significant using Chi-Square Test @ < 0.05 level

^b^No statistics are computed because variable is a constant

Crown failure during follow up intervals (Table 6) was clinically evaluated by visual assessment according to the US Public Health Service “USPHS”, Alpha criteria rating system [12]. From base line until the three months follow up, no failures was noticed in both groups. In the zirconia crown group, two crowns were lost completely in 6 and 12 months follow up due to trauma caused by falling of patients and hitting hard objects.

**Table 6:** Restoration failure evaluation

Chi-square test		Zirconia crowns	Strip crowns	p-value
*Restoration failure*				
At 3 months	Crowns appear normal, no cracks, chips, or fractures	60 (100.0%)	60 (100.0%)	N/A^c^
At 6 months	Crowns appears normal, no cracks, chips, or fractures	59 (98.3%)	52 (86.7%)	0.024^a^
	Small but noticeable area of loss of material	0 (0.0%)	7 (11.7%)	
	Large loss of crown	0 (0.0%)	1 (1.7%)	
	Complete loss of crown	1(1.7%)	0 (0.0%)	
At 12 months	Crowns appears normal no cracks, chips, or fractures	58 (98.3%)	37 (61.7%)	< 0.001^a^
	Small but noticeable area of loss of material	0 (0.0%)	18 (30.0%)	
	Large loss of crown	0 (0.0%)	5 (8.3%)	
	Complete loss of crown	1 (1.7%)	0 (0.0%)	

Friedman test	Mean rank			
	Total cases	Zirconia crown	Strip crown	
Baseline	2.37	2.49	2.24	
At 3 months	2.37	2.49	2.24	
At 6 months	2.5	2.49	2.51	
At 12 moths	2.77	2.53	3.01	
p-value	< 0.001^b^	0.392	< 0.001^b^	

^a^Significant using Chi-Square Test @ < 0.05 level

^b^Significant using Friedman Test @ < 0.05 level

^c^No statistics are computed because variable is a constant

On the other hand, failure was more in the strip crown group as 11.7% showed small but noticeable area of material marginal loss and 1.7% presented with large loss of crown during the 6 months follow up (*p* < 0.024). Moreover, this loss increased with time to reach 30% small but noticeable area of loss of material and 8.3% large loss of crown by the 12 months follow up (*p* < 0.001).

## Tooth wear evaluation

**Fig. 3:**
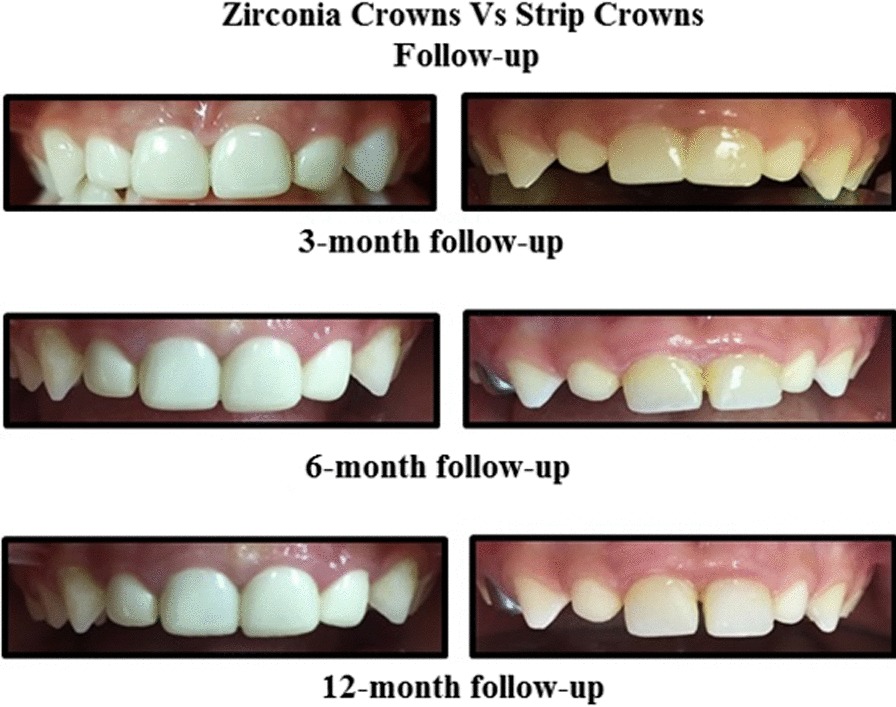
Intra-oral photographs showing the treatment of teeth no. 52, 51, 61, 62 by zirconia crowns (on the left), and teeth no. 52, 51, 61, 62 by Strip crowns (on the right) at different follow up intervals (3 months, 6 months and 12 months)

Tooth wear was evaluated (Table 7) according to Smith and Knight Tooth Wear Index [13, 14]. The incisal and labial surfaces of the teeth opposing the full-coronal restorations were clinically observed for any sign of abrasion. Seven teeth (11.7%) opposing to zirconia crowns showed loss of enamel surface characteristics and minimal loss of contour (*p* < 0.02), Fig. 3.

**Table 7:** Tooth wear evaluation

Chi-square test		Zirconia crowns	Strip crowns	*p*-value
*Tooth wear of opposing*				
At 3 months	No loss of enamel surface characteristics, no loss of contour	60 (100.0%)	60 (100.0%)	N/A^c^
At 6 months	No loss of enamel surface characteristics, no loss of contour	60 (100.0%)	60 (100.0%)	N/A^c^
At 12 months	No loss of enamel surface characteristics, no loss of contour	53 (88.3%)	60 (100.0%)	0.020^a^
	Loss of enamel surface characteristics, minimal loss of contour	7 (11.7%)	0 (0.0%)	

Friedman test	Mean rank			
	Total cases	Zirconia crown	Strip crown	
Baseline	2.47	2.44	2.50	
At 3 months	2.47	2.44	2.50	
At 6 months	2.47	2.44	2.50	
At 12 moths	2.59	2.68	2.50	
p-value	< 0.001^b^	< 0.001^b^	N/A^c^	

^a^Significant using Chi-Square Test @ < 0.05 level

^b^Significant using Friedman Test @ < 0.05 level

^c^No statistics are computed because variable is a constant

## Discussion

In evaluation of gingival health, this study shows better gingival response in zirconia crowns which can be explained by the fact that zirconia is biocompatible and possesses a polished and smooth surface leading to less plaque accumulation and hence less gingival irritation [16–18]. This is in accord with results reported in 2014 by Walia et al. [19] who evaluated anterior primary crowns for 129 patients (aged 3 to 5 years old). Their study revealed that zirconia crowns showed improved gingival health, while the other crowns (Composite strip crowns and pre-veneered SSCs) displayed more gingival inflammation. Another recent retrospective study by Holsinger et al. [20] assessing 57 primary anterior teeth treated with zirconia showed significant healthy gingiva in relation to these crowns.

A retrospective study done in 2003 by Kupietzky et al. [9] included 112 composite resin strip crowns found that 43% of the restored teeth showed gingival irritations around the crowns. These findings could be explained as the gingival health of teeth restored with composite strip crowns can be affected by tooth preparation and finishing [21, 22]. Unfortunately, upon reviewing the literature there were no sufficient data with regards to gingival response related to primary teeth restored by composite resin strip crowns. Padbury in 2003 [23], suggested placement of the strip crown margin supra gingivally to reduce gingival inflammation. Despite this recommendation being clinically logical, it is considered not applicable in most cases as it will result in poor aesthetics and appearance.

In agreement with our study, Walia et al. [19] who assessed anterior primary crowns for 129 patients (aged between 3 to 5 years old) also reported that zirconia crowns showed improved gingival health due to less plaque accumulation when compared to composite strip crowns and pre-veneered SSCs.

Our data showed that none of the teeth covered with zirconia crowns showed recurrent caries during the entire follow up. In contrast, teeth restored with composite resin strip crowns showed that 6.7% developed recurrent caries in the 12-months follow up. The lack of adequate preventive measures could have contributed in caries recurrence in our community. Important factors that influence development of caries are poor oral hygiene and high cariogenic diet consumed by the patients included in our study.

A recent study by Holsinger et al. [20] reported results similar to our study’s in their evaluation of 57 crowns treated with zirconia for primary anterior teeth in 18 patients. Their study showed no recurrent caries after a follow-up period of 24 months. Talebi et al. [24] evaluated the drawbacks of anterior primary crown restorations in 38 primary anterior teeth of 12 patients aged 3–5 years diagnosed with early childhood caries (ECC). Their results showed recurrent carious lesions at three and 12 months. One case displayed recurrent carious lesions around the margins of the restoration at the 3-months follow-up. While in eight teeth, the secondary caries occurred at the 12-month follow up over the boundaries of the restoration. Johnsen et al. [25] stated that patients diagnosed with ECC had higher tendency to develop recurrent caries after treatment. Another study done in 2000 by Almeida et al., found that young patients having ECC who were managed under general anesthesia to receive resin composite strip crown restorations exhibited significantly higher caries rates versus the control group who were caries free originally.

The greater restoration failure of the composite strip crowns in this study may be explained by the fact that treatment was done under nitrous oxide sedation and physical restrains to manage the children behavior. Eidelman et al. [26] reported that improved results for strip crowns were found in cases done under general anesthesia than those done under sedation. General anesthesia allows treatment to be rendered under theoretically optimal conditions; implying outcomes would be more successful. Success rate between 80 and 88% were found in the studies done by Kupietzky et al. [9]; Waggoner et al. [22]; Ram and Fuks [27]. High failure rate of 51% over a period of two years was seen in a study by Tate et al. [28] where strip crowns were placed under general anesthesia and endodontically treated teeth were included as well. Endodontic treatment can also affect the overall retention as these teeth are usually more damaged as mentioned by Kupietzky et al. [29].

Regarding the zirconia crowns, the success rate in this study was 98.3% by the end of the 12 months follow up. Only two crowns failed due to trauma. Current research on the clinical success of prefabricated primary zirconia crowns for primary incisors is still limited. Walia et al. [19] reported the retention rate of zirconia crowns as 100% after 6 months. These crowns have no facial upper structure, as they are made up of solid zirconia leading to no chance of facial veneer fracture as stated by Manicone et al. [30]. The flexural strength of zirconia oxide materials has been reported to be in the range of 900 to 1,100 MPa. This is approximately twice as strong as alumina oxide ceramics currently in the market and five times greater than standard glass ceramics [30]. Another important property is their fracture toughness making them perdurable and a highly strong restoration [31].

Tooth wear in this study was evaluated according to the Smith and Knight Tooth Wear Index [13, 14]. Seven teeth accounting for 11.7% of teeth opposing to zirconia crowns showed loss of enamel surface, minimal loss of contour compared to 100% no loss of enamel surface characteristics in strip crown group. This results is in agreement with Walia et al. [19] who found four opposing primary teeth out of 38 zirconia crowns having loss of enamel surface characteristics and minimal loss of contour.

Although one of the inclusion criteria was selecting patients with positive behaviour, some children occasionally became uncooperative due to prolonged procedure. Another limitation was the high cost of Zirconia crowns.

This study adds significant value to the literature with regards to the clinical performance of zirconia crowns in anterior primary molars. Although the zirconia crowns are considered expensive in comparison to strip crowns, we should take into consideration the high failure rate of strip crowns and the need to repeat dental visits and re-treatment of failed strip crowns. This fact may make the zirconia crowns cost effective after all as it has high success rate and minimal need for re-treatment.

## Limitations

Although one of the inclusion criteria was choosing patients with positive behaviour, in few cases children became uncooperative due to prolonged procedure. Another limitation was the high cost of Zirconia crowns.

## Recommendations

We recommend longer follow-up studies on zirconia crowns with inclusion of more evaluation parameters such as patients’ satisfaction especially with high cost of zirconia crowns, ease of handling, as well as other variables.

## Conclusions

Based on our data we can conclude that overtime teeth covered with zirconia crowns show better gingival health and less bleeding, plaque accumulation as well as less loss of material. On the other hand, zirconia can cause more loss of opposing tooth structure.

## Data Availability

The datasets used and/or analysed during the current study are available from the corresponding author on reasonable request.

## Abbreviations

KAUFD: King Abdulaziz University, Faculty of Dentistry
ECC: Early childhood caries
S-ECC: Severe early childhood caries
SSC: Stainless steel crowns
